# Supplementary figures and images for: Renalase is a novel tissue and serological biomarker in pancreatic ductal adenocarcinoma
Source: PLoS One. 2021 Sep 29;16(9):e0250539. doi: 10.1371/journal.pone.0250539 (PMC8480607; doi:10.1371/journal.pone.0250539)

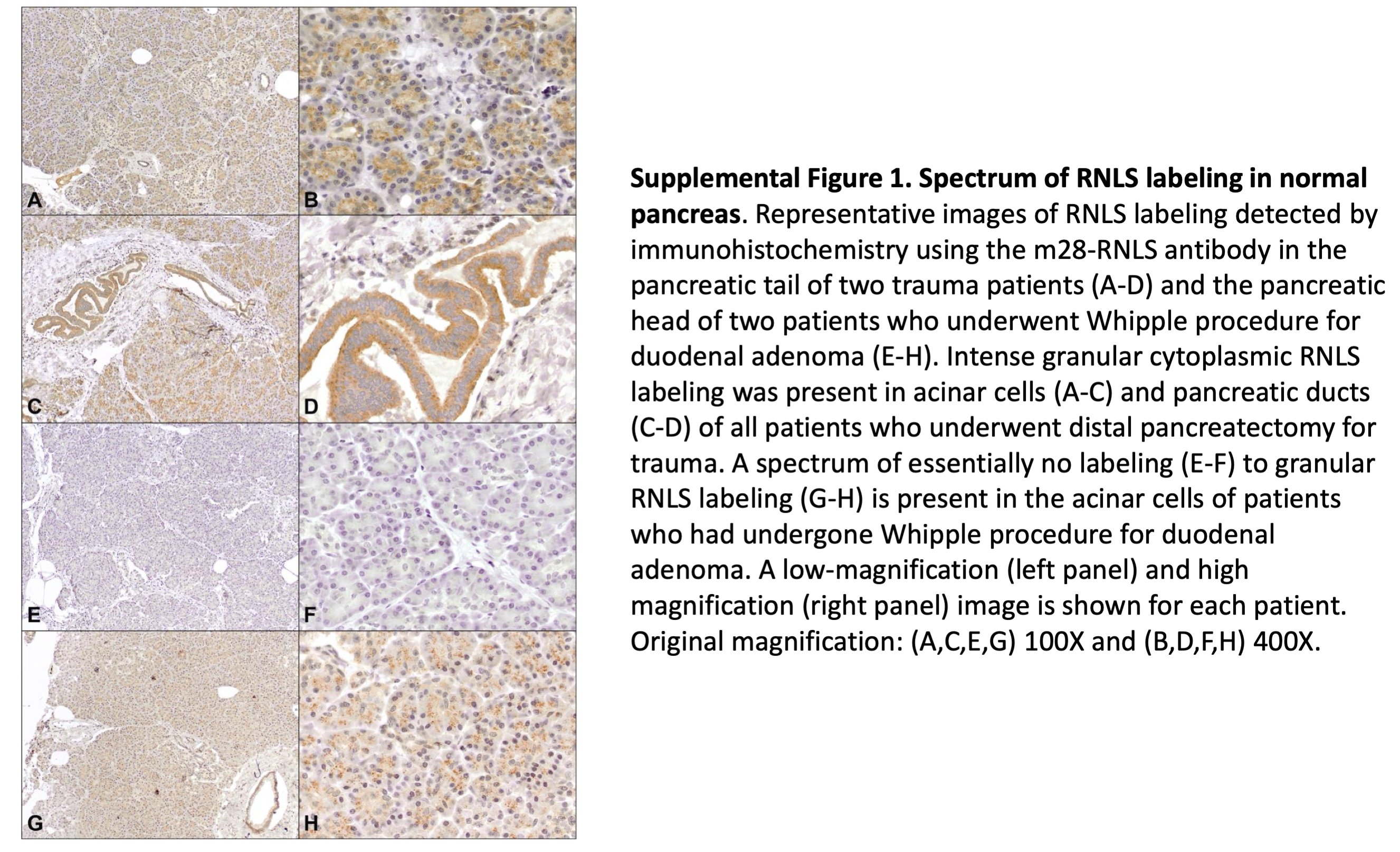

Supplement: S1 Fig — (TIFF) [file pone.0250539.s001.tiff]

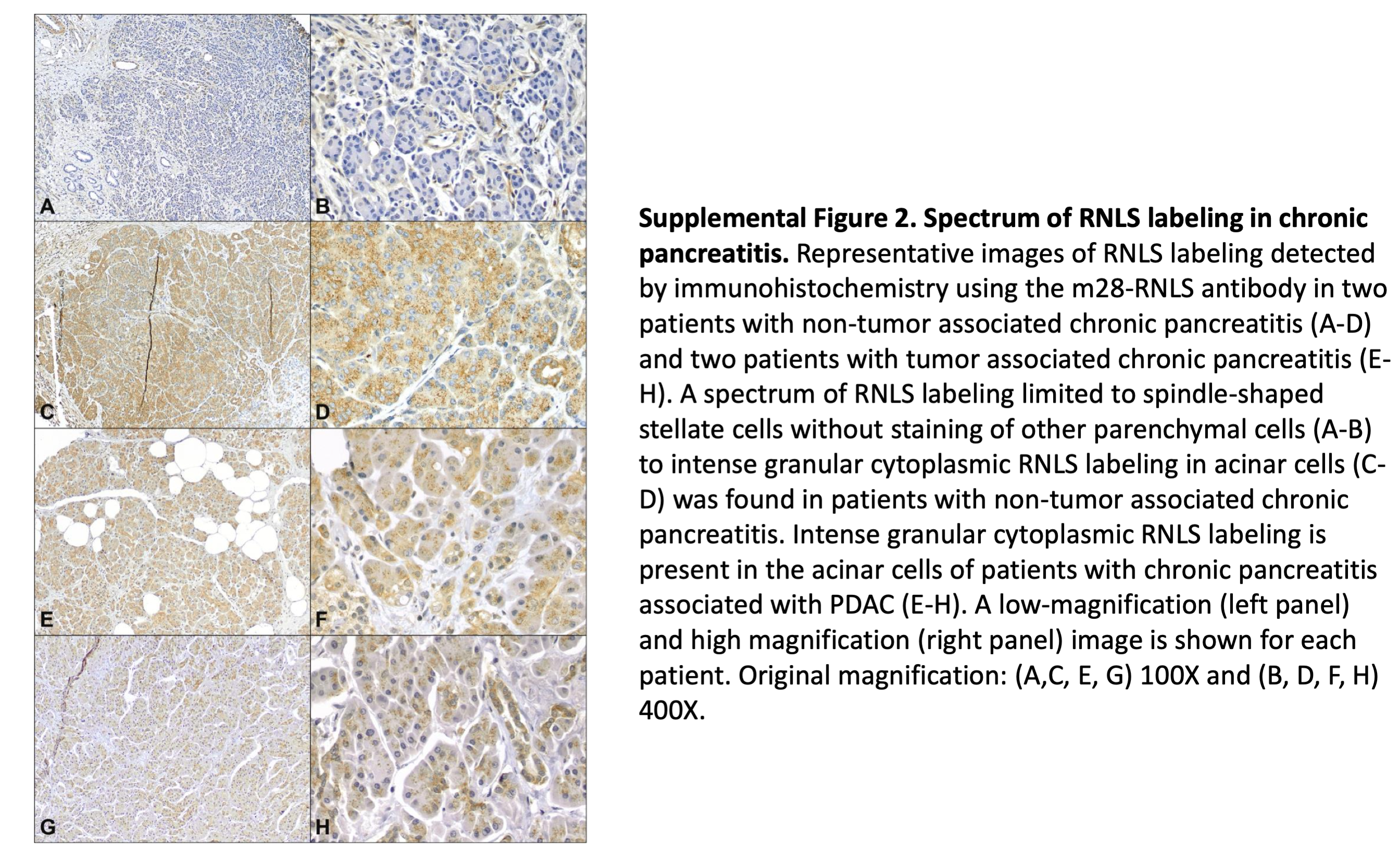

Supplement: S2 Fig — (TIFF) [file pone.0250539.s002.tiff]

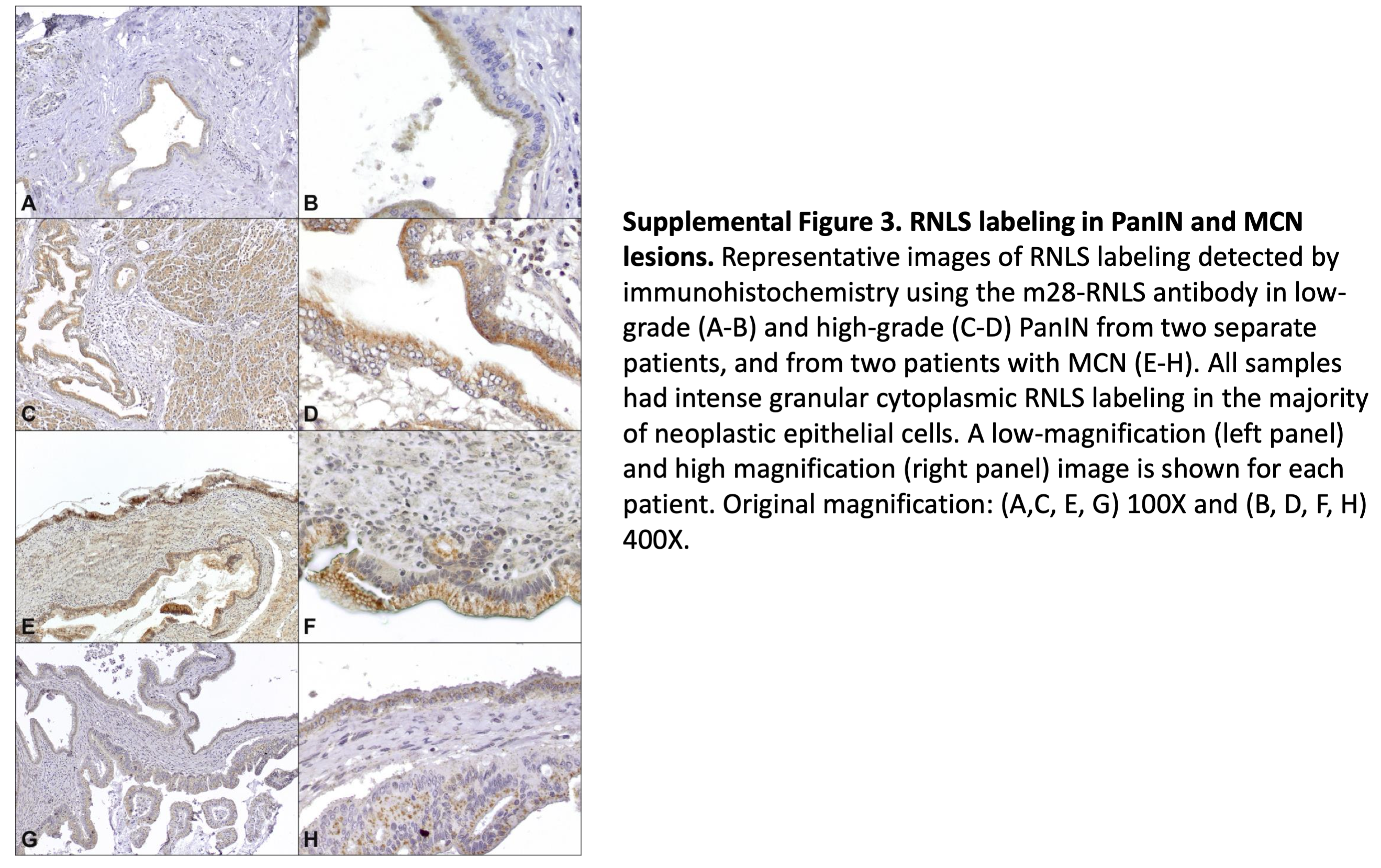

Supplement: S3 Fig — (TIFF) [file pone.0250539.s003.tiff]

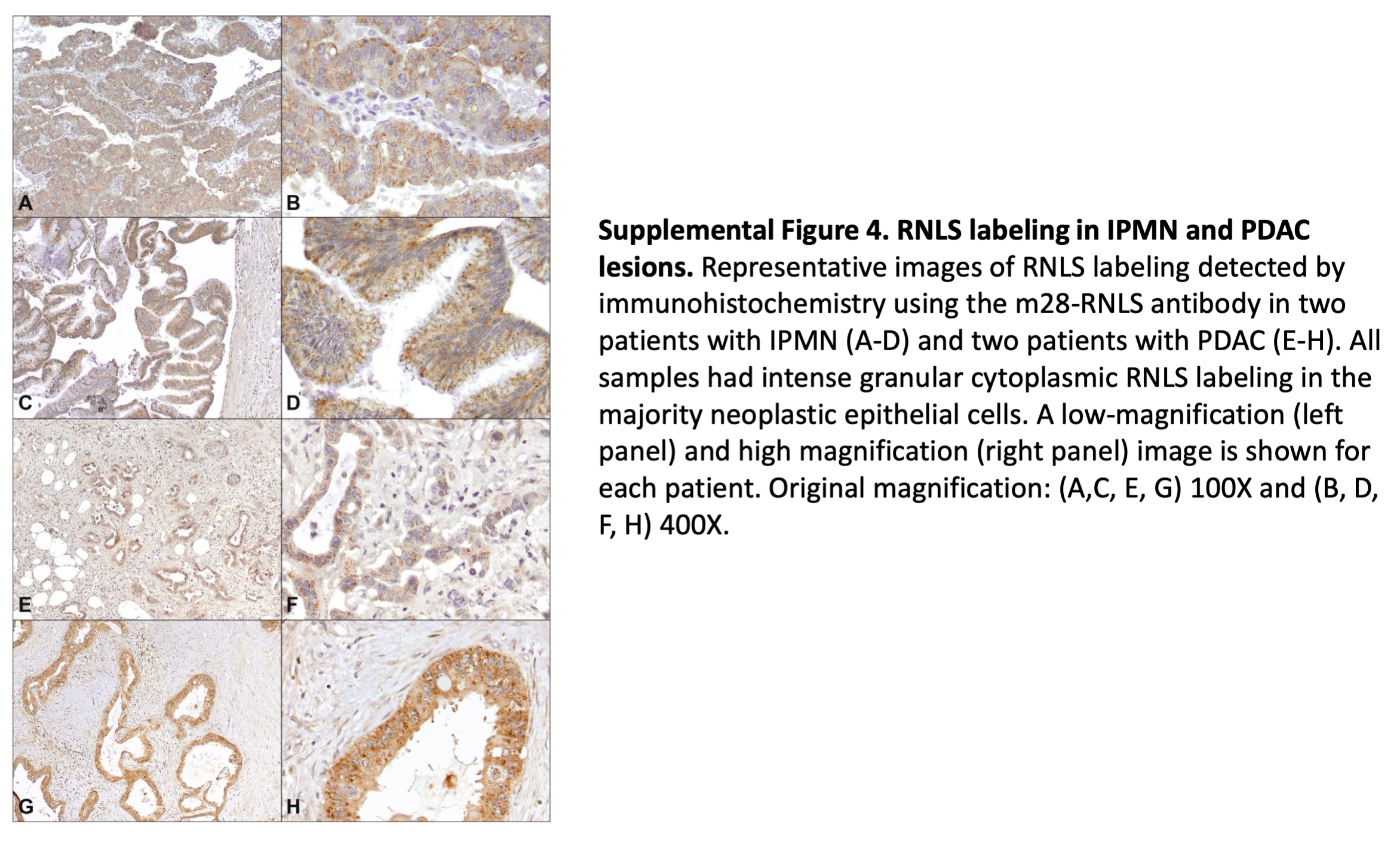

Supplement: S4 Fig — (TIFF) [file pone.0250539.s004.tiff]

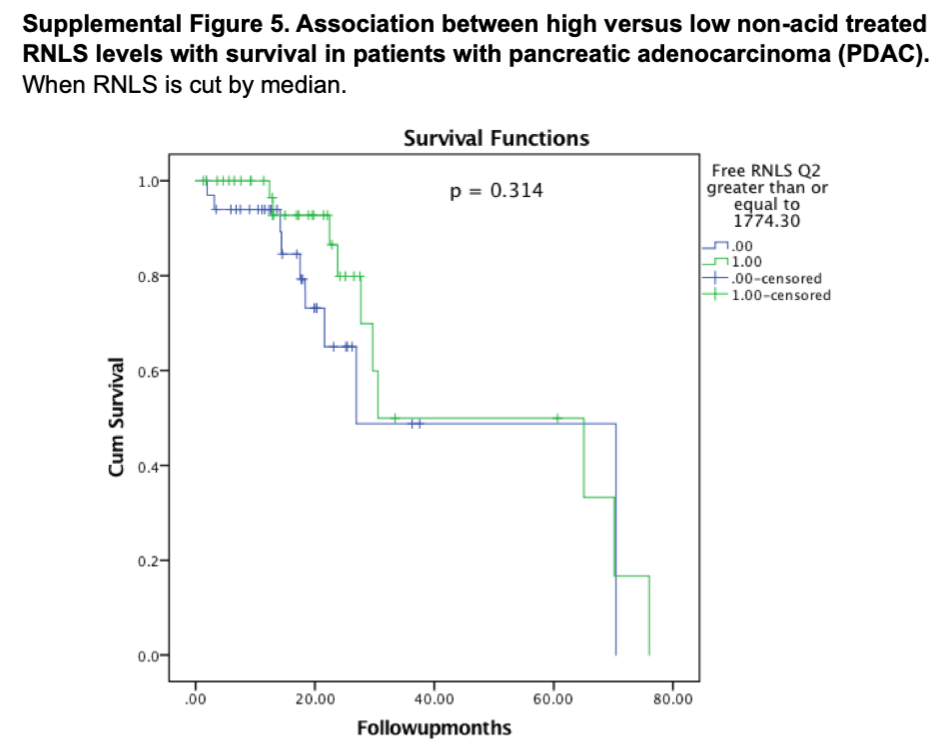

Supplement: S5 Fig — (TIFF) [file pone.0250539.s005.tiff]

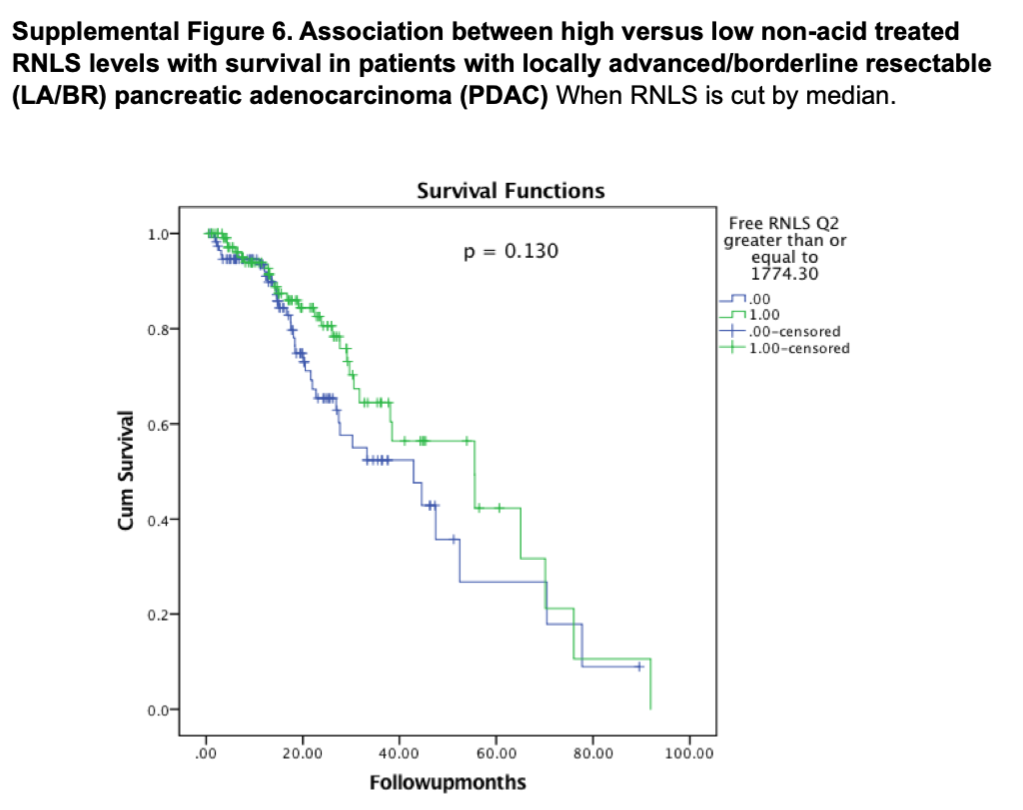

Supplement: S6 Fig — (TIFF) [file pone.0250539.s006.tiff]

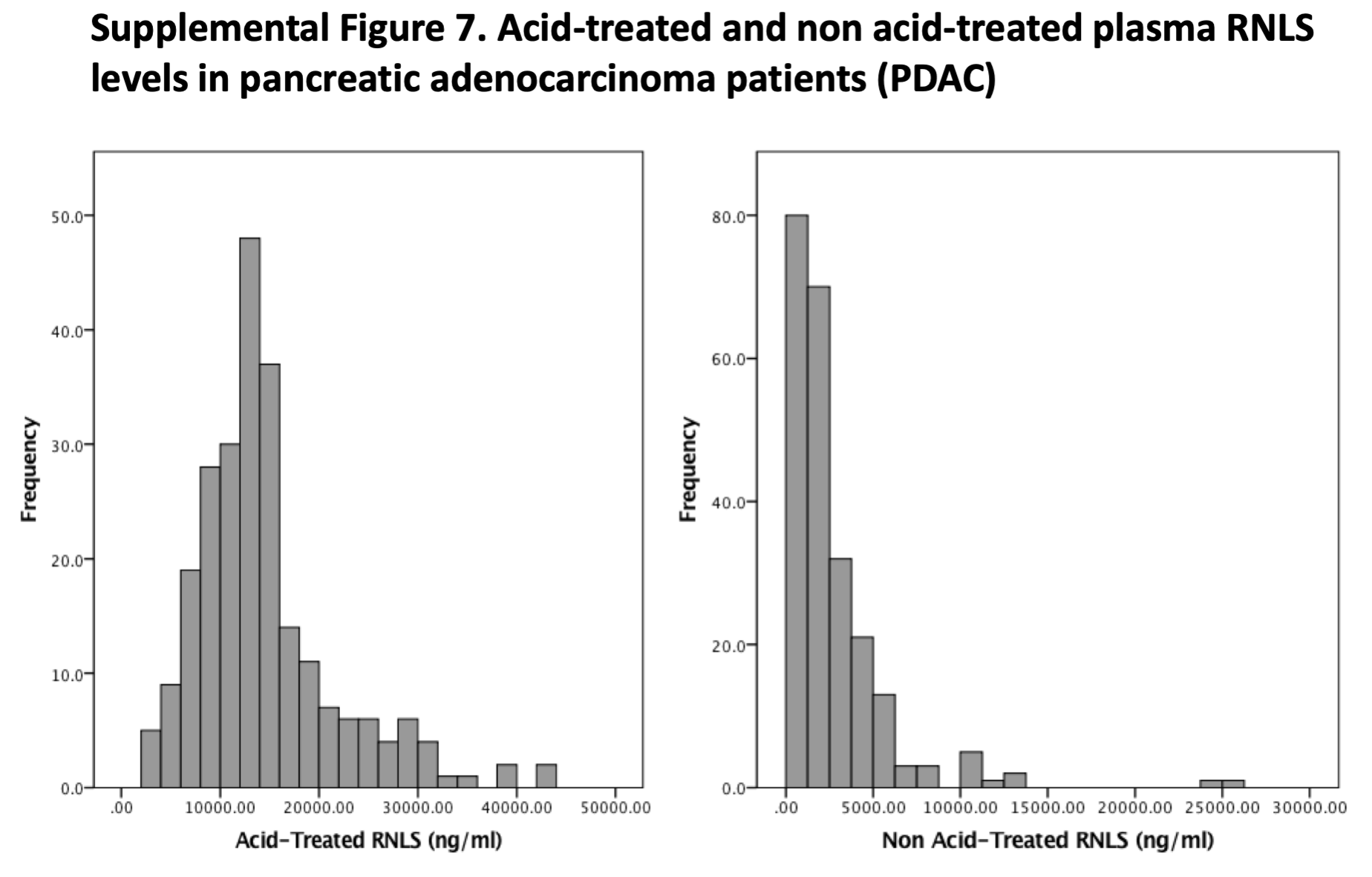

Supplement: S7 Fig — (TIFF) [file pone.0250539.s007.tiff]

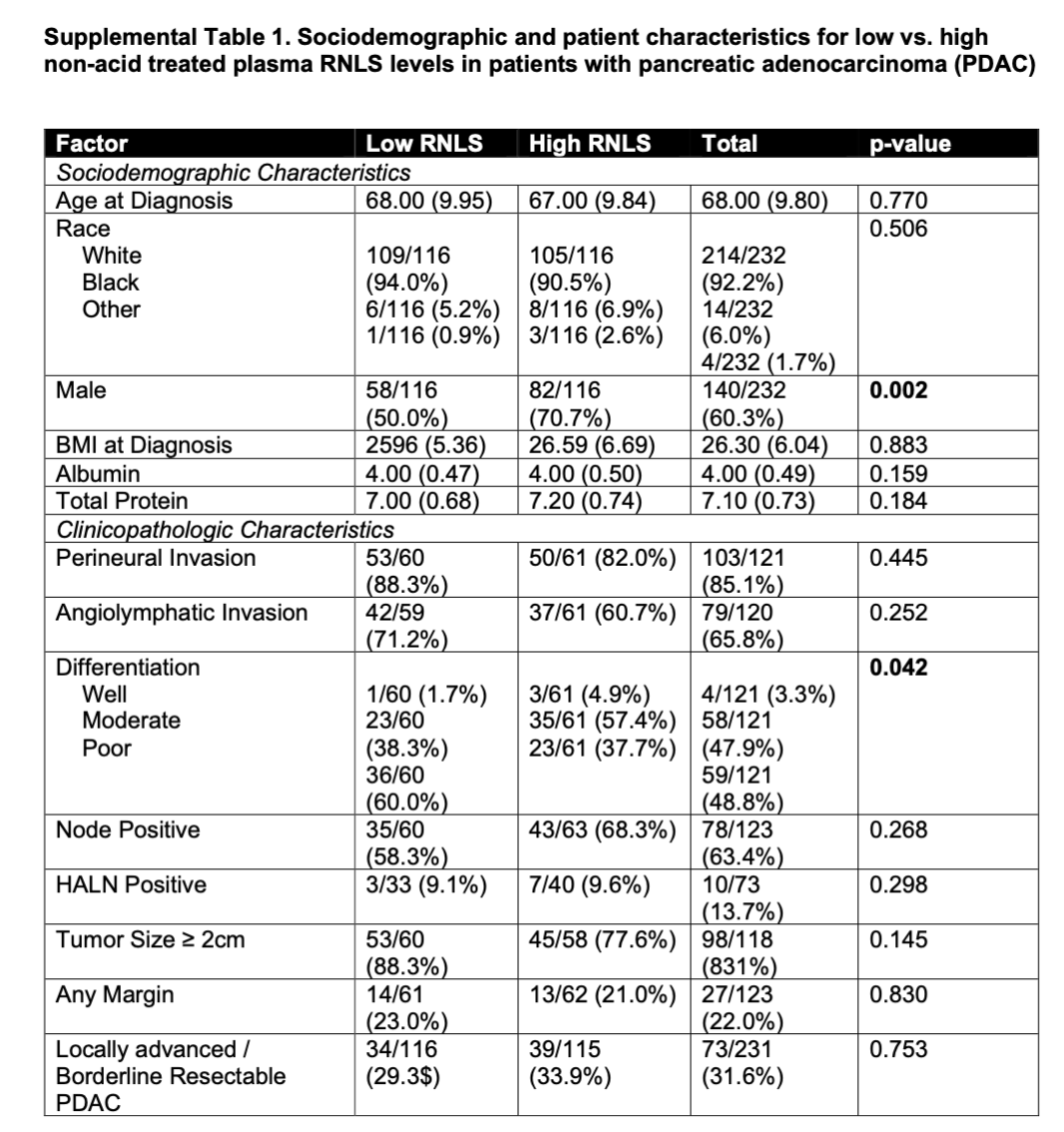

Supplement: S1 Table — (TIFF) [file pone.0250539.s008.tiff]
